# Enhancing the Mechanical and Adhesive Properties of Polyurethane Adhesives with Propylene Oxide-Modified Ethylenediamine (PPO-EDA)

**DOI:** 10.3390/polym17020231

**Published:** 2025-01-17

**Authors:** Nam Gyu Jang, Tran Quang Linh, Mai Toan, Kiok Kwon, Seunghan Shin

**Affiliations:** 1 Green Chemistry & Materials Group, Korea Institute of Industrial Technology (KITECH), Cheonan 31056, Republic of Korea; cnoo7546@korgc.com (N.G.J.); linhtran@kitech.re.kr (T.Q.L.); maitoan@kitech.re.kr (M.T.); kioks@kitech.re.kr (K.K.); 2 Department of Green Process and Energy System Engineering, University of Science & Technology (UST), Daejeon 34113, Republic of Korea

**Keywords:** polyurethane adhesive, PPO-EDA, crosslinker, mechanical properties, lap shear strength

## Abstract

This study explores the use of propylene oxide-modified ethylenediamine (PPO-EDA) as a novel crosslinker and chain extender in polyurethane (PU) adhesives. PPO-EDA was synthesized and compared with *N*,*N’*-dimethylethylenediamine (DMEDA) to assess its impact on mechanical properties and adhesion performance. Key parameters such as NCO conversion, tensile strength, and lap shear strength were thoroughly evaluated. The results demonstrated that incorporating PPO-EDA significantly improved NCO conversion and crosslink density, leading to notable enhancements in tensile strength and elastic modulus compared to DMEDA. Lap shear tests further revealed superior adhesion performance in PPO-EDA-modified PU adhesives, particularly on amine silane-treated steel substrates, where lap shear strength consistently outperformed other samples. This improved performance was attributed to PPO-EDA’s dual role as a chain extender and crosslinker, which strengthened the adhesive’s structural integrity. This study underscores the effectiveness of PPO-EDA as a modifier for enhancing both mechanical and adhesive properties in PU-based adhesives, offering a promising solution for optimizing high-performance adhesives in automotive and industrial applications.

## 1. Introduction

Polyurethane (PU) materials can be categorized into flexible and rigid foams, elastomers, coatings, sealants, and adhesives, depending on the ratio and type of components used. Flexible PU foams are widely utilized in the furniture and bedding industries, while rigid foams are commonly employed in insulation applications due to their high thermal resistance [1]. Elastomeric PUs are favoured for applications requiring abrasion resistance and elasticity, such as wheels, tires, and conveyor belts [2]. In coatings, sealants, and adhesives, PU formulations offer versatility, durability, and chemical resistance, finding use across a range of industrial and consumer products [3].

In structural bonding, PU adhesives have gained prominence in automotive and aerospace applications, where both high strength and flexibility are demanded. They provide durable bonds capable of withstanding harsh conditions, including vibration, impact, and temperature fluctuations [4]. By replacing mechanical fasteners and enhancing corrosion resistance, these adhesives help reduce vehicle weight, improve fuel efficiency, and streamline manufacturing processes [5,6,7]. Among various adhesive systems, PU adhesives are particularly valued for their excellent cohesive strength and flexibility, making them suitable for bonding dissimilar materials under high-stress conditions [8].

PU adhesives are typically formed through the reaction of diisocyanates with polyols, and their final properties are greatly influenced by the types of isocyanates and polyols used, as well as the reaction conditions [9]. Furthermore, catalysts such as tertiary amines or organometallic compounds are often employed to accelerate these reactions and ensure efficient polymerization. Recent advances in greener catalytic technologies and biodegradation strategies have shown promise in reducing toxicity and environmental impact in PU systems [10,11,12]. For instance, Levent et al. reported the effective replacement of organotin compounds with bismuth octoate and neodecanoate, where lithium carboxylate enhanced catalytic activity [10]. Sun et al. introduced a zirconium-based catalyst for sustainably processing PU covalent adaptable networks (CANs) [11]. Meanwhile, Olivito et al. demonstrated the feasibility of biodegradable PU production from renewable resources via a prepolymer method extended with 2,5-bis(hydroxymethyl)furan [12]. These studies highlight an evolving focus on both performance and ecological considerations in PU research.

Amine compounds are particularly important in PU formulations because they act as chain extenders or crosslinkers, reacting with isocyanate groups to form urea linkages that enhance the adhesive’s strength and durability [13,14,15]. The introduction of novel crosslinkers and chain extenders can further enhance the adhesive’s performance by increasing crosslink density and improving molecular structure. For example, *N*,*N′*-dimethylethylenediamine (DMEDA) effectively extends polymer chains by forming linear urea linkages but does not substantially raise crosslink density [16]. While DMEDA can improve tensile strength to some extent, its role is limited to chain extension, which may not sufficiently optimize the adhesive’s mechanical properties for demanding applications.

In contrast, propylene oxide-modified ethylenediamine (PPO-EDA) introduces both secondary amine (NH) and secondary hydroxyl (OH) groups into the PU network. These functional groups allow PPO-EDA to act both as a chain extender and a crosslinker, reacting with isocyanate groups to form urea and urethane linkages, respectively. This dual functionality could significantly increase the crosslink density of the adhesive, potentially leading to enhanced mechanical performance. Moreover, the ethylene linkages in PPO-EDA may contribute to greater segmental mobility within the polymer chains [17,18], possibly improving flexibility despite increased crosslinking.

Despite these potential advantages, the effects of PPO-EDA on the mechanical properties and adhesion performance of PU adhesives have not been thoroughly investigated. Therefore, the present study focuses on synthesizing PPO-EDA and evaluating its role as a novel crosslinker and chain extender in PU adhesives. The performance of PPO-EDA is compared with that of DMEDA to assess their influence on key properties, including NCO conversion, tensile strength, and lap shear strength. By examining the distinct roles of DMEDA and PPO-EDA in the PU adhesive structure, this study aims to contribute to the development of advanced structural adhesives with an optimized balance between stiffness and flexibility for high-performance applications in the automotive industry.

## 2. Experiments

### 2.1. Materials

Propylene oxide (PPO, 99%) and ethylene diamine (EDA, 98%) were sourced from Tokyo Chemical Industry Co., Ltd (Tokyo, Japan). Ethanol (EtOH, 99%); hexane (Hx, 98%), ethyl acetate (EA, 99.8%), dimethyl ether (DME, 99%), triethylamine (TEA, 99%), and *N*,*N′*-dimethylethylenediamine (DMEDA, 98%) were purchased from Sigma Aldrich (St. Louis, MO, USA). Further, 3-Aminopropyl trimethoxysilane (APS, ≥97%) and toluene (anhydrous, 99.8%) were supplied by Sigma Aldrich and used for the surface modification of the steel substrate.

The two-component PU-based structural adhesive was formulated using resin A and resin B, both provided by T&L (Yongin City, Republic of Korea). Resin A contained polycaprolactone polyol (PLACCEL 410, Daicel, Japan), polycarbonate diol (Ravecarb 103, Caffaro industrie SpA, Torviscosa, Italy), and 1,3-butanediol. Resin B consists of modified methylene diphenyl diisocyanate (Cosmonate LL, Kumho Mitsui Chemicals, Seoul, Republic of Korea), polyester polyol (HP-1010, Zhejiang Hexin Science and Technology, China), and polyether polyol (GP-400, KPX Chemical, Seoul, Republic of Korea). The NCO/OH ratio of the PU adhesive was 1.084. Calcium carbonate (CaCO_3_, Omyacarb 10) was supplied by Omya (Oftringen, Switzerland), and the desiccant (SUPERSIV 3A) was purchased from Baltimore Innovations (Bourne End, UK).

### 2.2. Synthesis of 1,1’-(Ethane-1,2-Diylbis(Azanediyl))Bis(Propan-2-ol) (PPO-EDA)

**Scheme 1:**
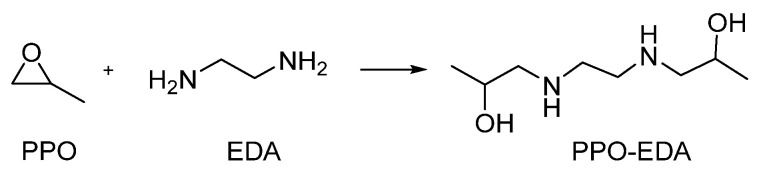
Synthesis of PPO-EDA.

PPO-EDA was synthesized following the procedure outlined in Scheme 1. In a nitrogen atmosphere, a 500 mL three-neck flask equipped with a mechanical stirrer was charged with EtOH (20 mL) and EDA (3.01 g, 0.05 mol). Propylene oxide (5.81 g, 0.1 mol) in EtOH (30 mL) was then added dropwise at room temperature using a syringe pump at a rate of 100 mL/h. Upon completion of the addition, the reaction was stirred for an additional 4 h. The reaction mixture obtained was crystallized at −20 °C, followed by filtration and additional washing with DME to yield a high-purity white powder. The final product was obtained with a yield of 41.0%.

### 2.3. Preparation of PU-Based Structural Adhesives 

The resin and hardener for two-component PU adhesives were prepared as outlined in Table 1. For the resin preparation, resin A (polyols), desiccant, CaCO_3_ and additive (DMEDA or PPO-EDA) were mixed and degassed by a paste mixer (ARV-310, Thinky, Tokyo, Japan) at 2000 rpm under 1.0 kPa for 3 min. The hardener, consisting of resin B (modified-MDI, polyols) and CaCO_3_, was prepared using the same method. Prior to use, the resin and hardener were mixed using a two-component cartridge-dispensing gun (Nordson EFD, Westlake, OH, USA).

### 2.4. Reaction Monitoring of PPO-EDA and Phenyl Isocyanate

To assess the reactivity of PPO-EDA with isocyanates at room temperature, phenyl isocyanate was used as a model compound. The reaction was conducted by adding PPO-EDA (0.5 g, 5.6 mmol) and THF (30 mL) to a round-bottomed flask and stirring at 300 rpm. Phenyl isocyanate (2.027 g, 33.6 mmol), dissolved in 10 mL of THF, was added dropwise over 1 h. The mixture was stirred for 24 h at 30 °C. The products were separated by column chromatography (Hx–EA = 1:4), dissolved in DMSO-d6, and the structure of the products was confirmed by ^1^H-NMR analysis.

### 2.5. Characterization

The ^1^H- and ^13^C-NMR spectra of the PPO-EDA were recorded using an FT-NMR spectrometer (300 MHz, Spectrospin 300, Bruker, Billerica, MA, USA), with CD_3_OD as the solvent. Fourier transform infrared spectroscopy (FT-IR) spectroscopy of the two-component PU adhesives was conducted using a NICOLET 6700 FT-IR spectrometer (Thermo Fisher Scientific, Waltham, MA, USA) to assess the NCO peak conversion. The PU adhesives were applied to KBr pellets, and the transmittance spectra were collected in the wavenumber range of 4000–500 cm^−1^, with a resolution of 4 cm^−1^. The transmittance data were converted to absorbance spectra using Thermo Fisher Scientific OMNIC software (version 9.8). 

Tensile tests were conducted according to the ASTM D638 (Type V) standard [19]. The PU adhesives were cast into silicone molds and cured for 24 h to prepare specimens with dimensions of 63.50 mm × 3.18 mm × 3.40 mm (length (l) × width (w) × thickness (t)). Tensile properties were measured using a universal testing machine (KDMT-156-10, Kyung Do Precision Co., Gyeong Ju City, Republic of Korea) at a crosshead speed of 0.5 mm/min.

The viscoelastic properties of the PU adhesives were analyzed using a dynamic mechanical analyzer (DMA, TT-DMA, Mettler-Toledo, Columbus, OH, USA). Specimens with dimensions of 20 mm × 2 mm × 1 mm (l × w × t) were tested in single cantilever mode. The temperature range was −100 to 100 °C with a heating at a rate of 10 °C/min, and the tests were conducted at a frequency of 1 Hz.

Lap shear tests followed the ISO 4587 standard method [20]. CR340 steel plates with dimensions of 25 mm × 25 mm × 1.6 mm (l × w × t) were used as substrates. PU adhesives were applied to a 25 mm × 12.5 mm (l × w) area of the plates with a controlled thickness using 0.2 mm glass beads. The overlapped plates were clamped and cured at room temperature for 24 h. The lap shear strength was measured using a universal testing machine (KDMT-156-10, Kyung Do Precision Co., Gyeong Ju City, Republic of Korea) at a crosshead speed of 5 mm/min.

After the lap shear tests, the fracture surfaces of the adhesive joints were examined to determine the failure mode. High-resolution photographs of the fractured surfaces were captured using a smartphone camera (iPhone 12 Pro, Apple Inc., Cupertino, CA, USA). The images were taken under consistent ambient lighting conditions, with the camera lens positioned perpendicular to the fracture surface at a fixed distance of approximately 10 cm to ensure focus and clarity. No additional lenses or imaging accessories were used.

## 3. Results and Discussion

### 3.1. Synthesis of Epoxide-Modified EDA

**Figure 1:**
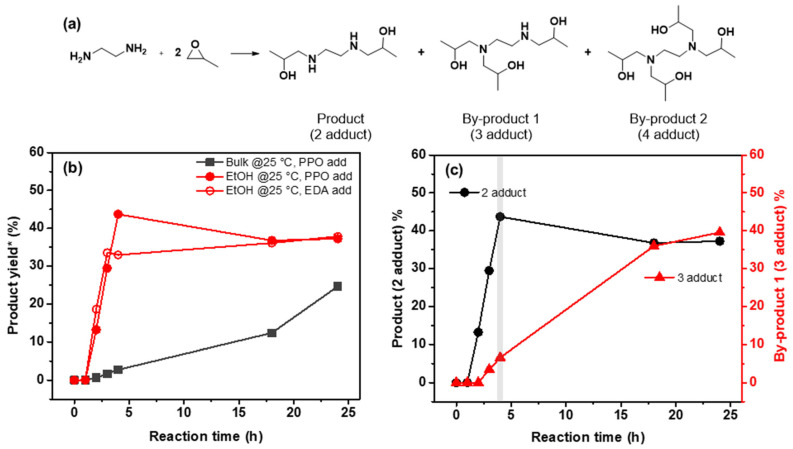
(**a**) Possible reaction products between propylene oxide and ethylenediamine, (**b**) effect of reaction conditions (solvent, reagent addition order) on product yield as a function of reaction time, and (**c**) yield of the main product and by-product 1 in ethanol. * Yields are calculated based on GC area percentage.

The synthesis of PPO-EDA was conducted based on reported method [21], the procedure was refined by adjusting critical parameters such as reactant concentration, reaction temperature, and solvent composition to enhance the overall yield. To optimize the reaction conditions between PPO and EDA, the reaction progress was monitored via GC. The primary product identified was 2-adduct, while 3-adduct and 4-adduct were detected as by-products (Figure 1a). To maximize the yield of the desired product, the reaction temperature was maintained at 25 °C while adjusting variables such as reaction medium (bulk or ethanol), the order of reagent addition (diamine or epoxide first), and reaction time.

As shown in Figure 1b, the use of ethanol as a solvent significantly improved the product yield compared to bulk reaction. While the order of reagent addition influenced the yield initially, this effect diminished over time. After 4 h at 25 °C in ethanol, the highest yield of the target product (41%) was obtained. However, as the reaction proceeded, the formation of the 3-adduct gradually reduced the target product yield (Figure 1c), as summarized in Table 2. Therefore, within our experimental conditions, the optimal conditions for synthesizing PPO-EDA were confirmed to be 4 h at 25 °C using ethanol as the solvent.

**Figure 2:**
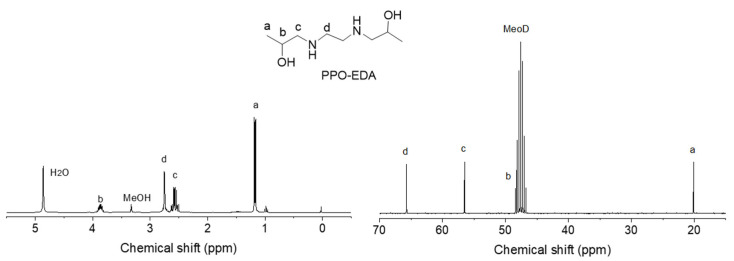
^1^H-NMR (**left**) and ^13^C-NMR (**right**) spectra of the target product, PPO-EDA.

The chemical structure of the target product, PPO-EDA, was verified using ^1^H- and ^13^C-NMR (Figure 2), and its melting and boiling points were determined to be 121 °C and 290 °C, respectively.

### 3.2. NCO Conversion of PU Adhesives

**Figure 3:**
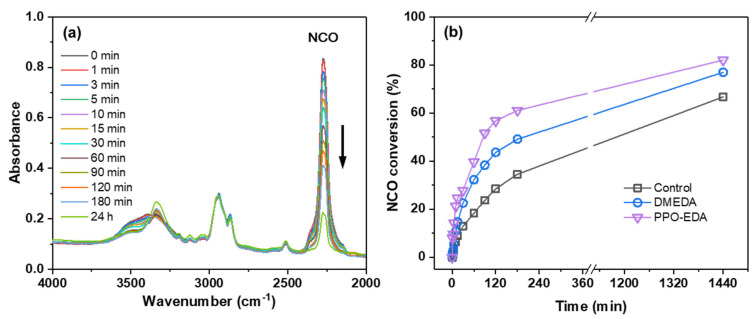
(**a**) FTIR spectra of PU adhesives over time, and (**b**) NCO conversion of PU adhesives containing different ethylenediamines as a function of reaction time.

Figure 3a shows the FTIR spectra of PU adhesives measured at room temperature over different curing times. As curing progressed, the OH stretching peak at 3500 cm^−1^ (from polyols) and the NCO stretching peak at 2250 cm^−1^ (associated with isocyanate groups) both decreased. This decrease indicates the formation of urethane and urea bonds through reactions with polyols and moisture [22,23,24,25,26]. Quantitative analysis of the NCO peak intensity revealed the following NCO conversion rates after 24 h: control (66.7%) < DMEDA (76.9%) < PPO-EDA (82.0%). These results are shown in Figure 3b.

**Figure 4:**
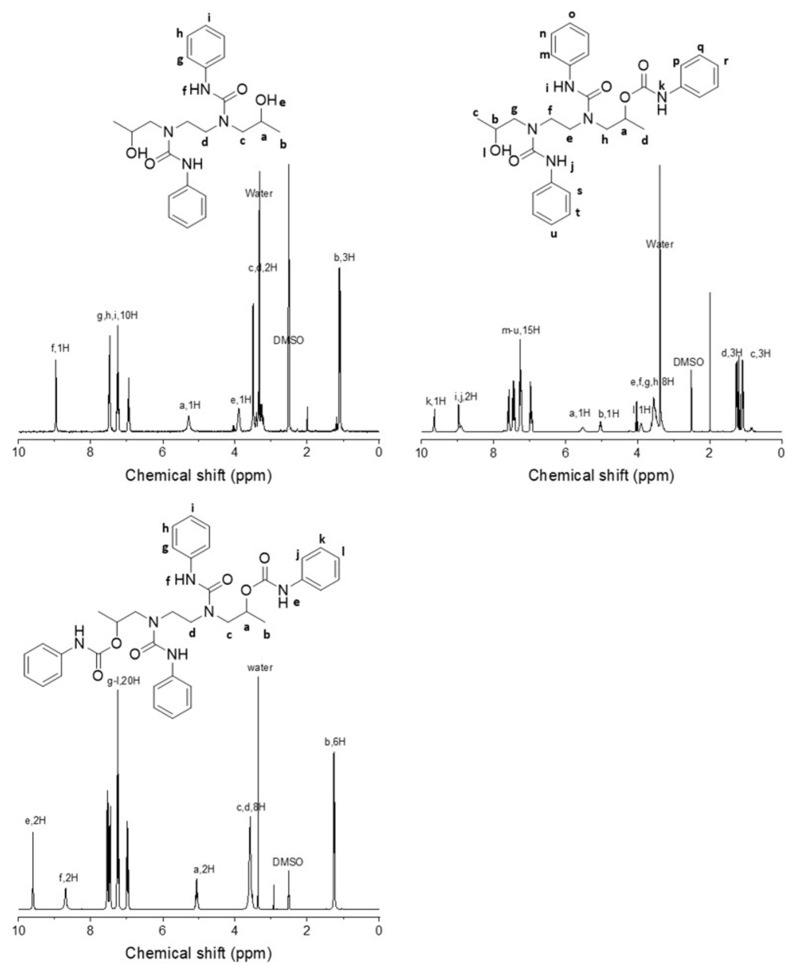
^1^H-NMR spectra of the reaction products of PPO-EDA with phenyl isocyanate.

The chemical structure of PPO-EDA contains two secondary hydroxyl groups and two secondary amine (NH) groups, which react with NCO groups to form urethane and urea bonds, respectively. Typically, the reactivity of functional groups with isocyanates follows the order of primary amine > secondary amine > primary alcohol > secondary alcohol. To confirm the reactivity of PPO-EDA’s secondary amine and hydroxyl groups with isocyanates, PPO-EDA was reacted with phenyl isocyanate at room temperature and analyzed over time using ^1^H-NMR (Figure 4). This reaction revealed the formation of di-, tri-, and tetra-adducts, confirming that PPO-EDA functions both as a chain extender and a crosslinker during PU adhesive curing.

### 3.3. Preparation and Structural Considerations of PU-Based Adhesives

**Figure 5:**
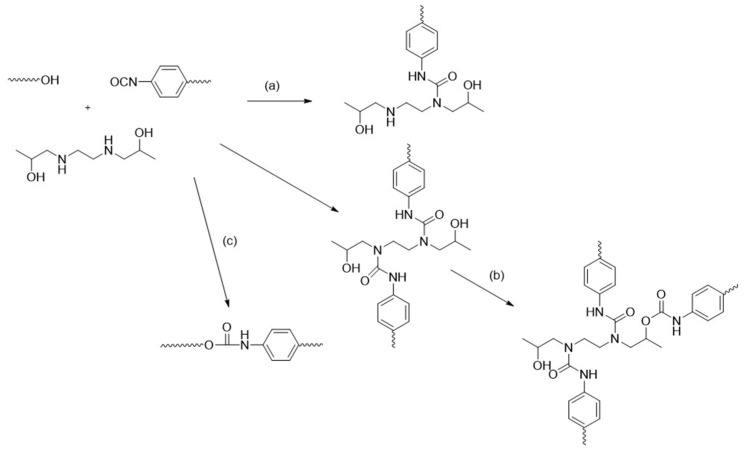
Synthetic pathway of PU-based structural adhesives showing urea and urethane linkages formed from PPO-EDA, polyols, and m-MDI.

Figure 5 illustrates the synthetic pathway for the PU-based structural adhesives prepared in this work. The resin component comprises polyols (tetra-ol and tri-ol), PPO-EDA, a desiccant, and CaCO_3_. The hardener contains modified methylene diphenyl diisocyanate (m-MDI). When these two components are blended, the secondary amine and hydroxyl groups of PPO-EDA rapidly react with the isocyanate moieties in m-MDI to form both urea and urethane linkages (Schemes (a) and (b)). Consequently, PPO-EDA acts as both a chain extender and a crosslinker, raising crosslink density while also introducing flexible segments through its ethylene backbone, thus enhancing the adhesive’s initial mechanical strength.

In contrast, the polyols primarily form the soft segment by reacting with m-MDI to create urethane linkages. However, because the tetra-ol and tri-ol are multifunctional, they also introduce a limited number of crosslink points. The comparatively long aliphatic segments in these polyols increase the inter-crosslink distance, which imparts enhanced segmental mobility and flexibility within the network. Consequently, PPO-EDA and multifunctional polyols together produce a hybrid network in which crosslink density is increased by PPO-EDA, yet flexibility is retained via aliphatic chains and secondary functionalities.

Overall, this strategy leverages both the rapid reactivity of PPO-EDA for immediate network formation and the polyols’ longer aliphatic segments for chain mobility. This interplay between urea and urethane linkages allows for increased stiffness without a proportional increase in *T_g_*, as demonstrated by the enhanced tensile and lap shear strengths discussed below.

### 3.4. Tensile Properties of PU Adhesives

**Figure 6:**
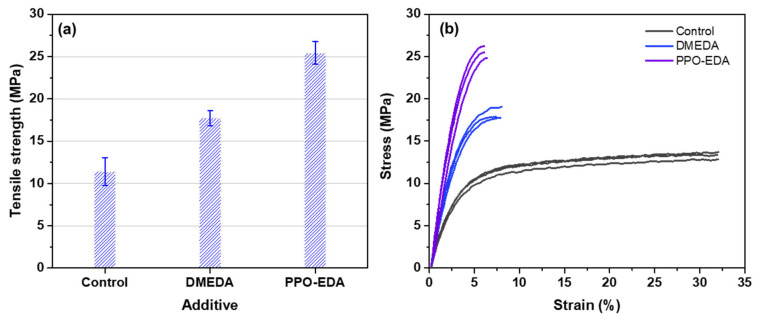
(**a**) Tensile strength and (**b**) stress–strain curves of PU adhesives with different additives, measured at a crosshead speed of 10 mm/min.

Figure 6a illustrates the tensile strength of the PU adhesives after 24 h of curing at room temperature. The addition of DMEDA increased the tensile strength, with PPO-EDA exhibiting the highest value among the samples. Stress–strain analysis (Figure 6b) showed that incorporating these additives decreased elongation and increased Young’s modulus, indicating enhanced the stiffness and rigidity of the PU adhesive [26,27].

These results are closely related to the NCO conversion (Figure 3). PU adhesives with higher NCO conversion typically exhibit superior tensile strength. Specifically, PPO-EDA significantly promoted urethane and urea bond formation, leading to increased tensile strength and elastic modulus, whereas DMEDA showed a comparatively lesser effect. The greater tensile strength observed with PPO-EDA is likely due not only to the increased formation of urea bonds—which are stronger and more rigid than urethane bonds—but also to its ability to act both as a chain extender and a crosslinker, thereby enhancing the crosslink density of the PU network. In contrast, DMEDA acts only as a chain extender and does not contribute to crosslinking.

**Figure 7:**
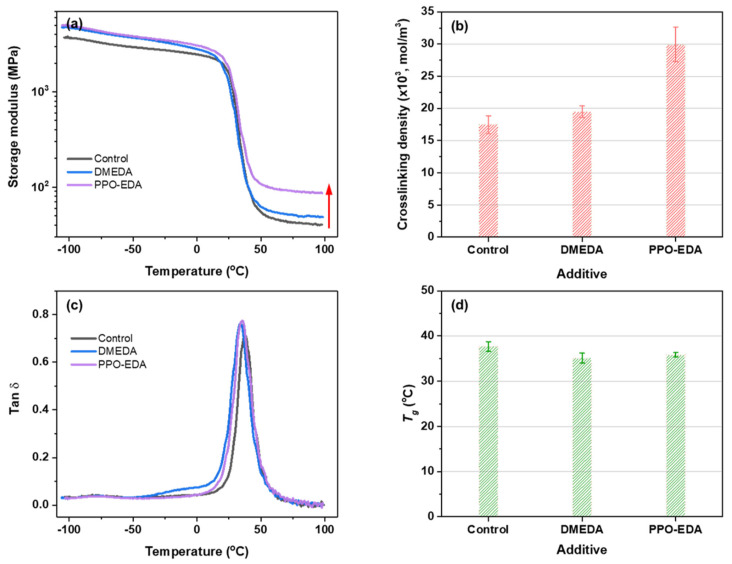
(**a**) Storage modulus, (**b**) crosslinking density, (**c**) loss tan delta, and (**d**) glass transition temperature of PU adhesives with various additives.

As shown in Figure 3, PPO-EDA exhibited a higher NCO conversion rate than DMEDA. This higher reactivity is attributed to the presence of both secondary NH and OH groups in PPO-EDA, which react with NCO groups to form urea and urethane linkages, respectively. The dual functionality of PPO-EDA enhances crosslink density and tensile strength in the PU adhesives. The crosslink density was calculated using the storage modulus at *T_g_* + 30 °C, according to the rubbery plateau theory [28]. Crosslink density (ν_e_) was calculated using below Equation (1).

(1) $$\nu_{e}=\frac{G^{\prime }}{RT}$$

where the storage modulus values (*G*′) are obtained in the rubbery plateau, *T* is temperature in degrees K, and *R* is the gas constant (8.314 Pa·m^3^/mol·K). The results are presented in Table 3 and Figure 7. PPO-EDA induced a higher crosslink density compared to DMEDA and the control (29.9 vs 19.5 vs 17.5 (×10^3^ mol/m^3^)), even though the *T_g_* values of the PU adhesives were similar (35.8 vs 35.1 vs 37.7 °C).

Typically, an increase in crosslink density leads to a rise in *T_g_*. However, in this case, *T_g_* slightly decreased, indicating the influence of other factors. The ethylene linkages in PPO-EDA introduce flexibility due to their aliphatic nature, allowing greater segmental mobility within the polymer chains. Additionally, the secondary NH and OH groups form relatively flexible bonds, further facilitating segmental motion and contributing to the lower *T_g_*. Although urea bonds create strong hydrogen bonds, they also preserve chain flexibility because of their linear structure, which can enhance chain mobility near *T_g_*. As a result, the flexibility provided by the ethylene linkages and secondary groups likely counteracts the rigidity from the increased crosslink density, leading to a slight reduction in *T_g_*.

### 3.5. Lap Shear Strength of PU Adhesives

**Figure 8:**
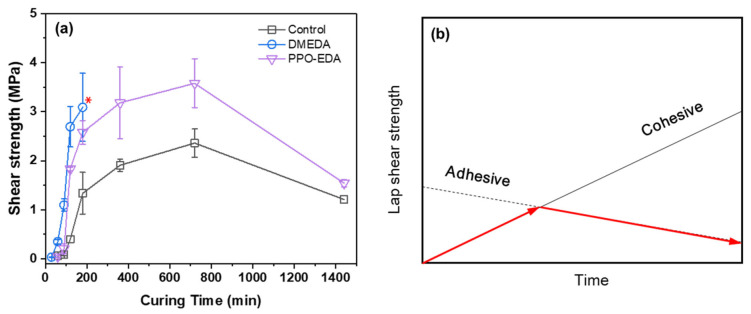
(**a**) Lap shear strength of PU adhesives with various additives as a function of curing time, and (**b**) schematic diagram illustrating the development of adhesive and cohesive forces in PU adhesives over curing time. The crosshead speed was 10 mm/min. * Indicates shear strength values too low to be measured beyond this curing time.

Figure 8a shows the lap shear strength of PU adhesives applied to pristine steel substrates over varying cure times. For both the control and the PPO-EDA-modified adhesives, the lap shear strength initially increases with cure time, reaches a peak, and then decreases. The DMEDA-modified adhesive shows an increase in lap shear strength up to 180 min, after which it decreases rapidly, preventing further testing. This behavior is attributed to a shift in failure mode; as the cure reaction progresses, the PU adhesive transitions from cohesive failure to interfacial failure on the pristine steel substrate.

Interfacial adhesion between PU adhesives and steel primarily relies on dipole–dipole interactions between adsorbed NCO groups and the steel surface, as well as covalent and hydrogen bonding between urethane groups and iron oxide [29]. In the early stages of curing, an abundance of NCO groups facilitates strong chemical bonding with the steel surface, enhancing adhesion. However, as curing progresses, NCO groups are consumed through reactions with polyols and additives like DMEDA and PPO-EDA, reducing their availability for interfacial bonding. Additionally, increased viscosity and crosslink density hinder the mobility of reactive groups, diminishing interactions with surface hydroxyl groups on the steel. Consequently, while the cohesive energy within PU adhesive increases, the adhesive energy at the interface decreases over time (Figure 8b).

**Figure 9:**
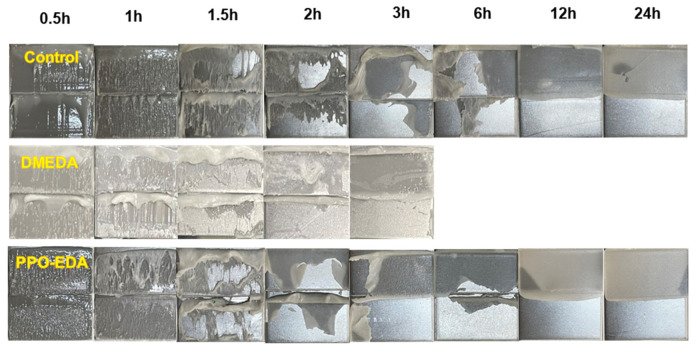
Photo images of the fractured surface of PU adhesives after lap shear testing.

This phenomenon is confirmed by observing the failure modes. Surface analysis of the fractured PU adhesive (Figure 9) revealed that as curing advances, the failure mode shifts from cohesive to mixed and eventually to adhesive failure. This indicates that despite the increased cohesion from additives like DMEDA and PPO-EDA, the lap shear results on pristine steel substrates do not reflect this improvement due to interfacial failure.

**Figure 10:**
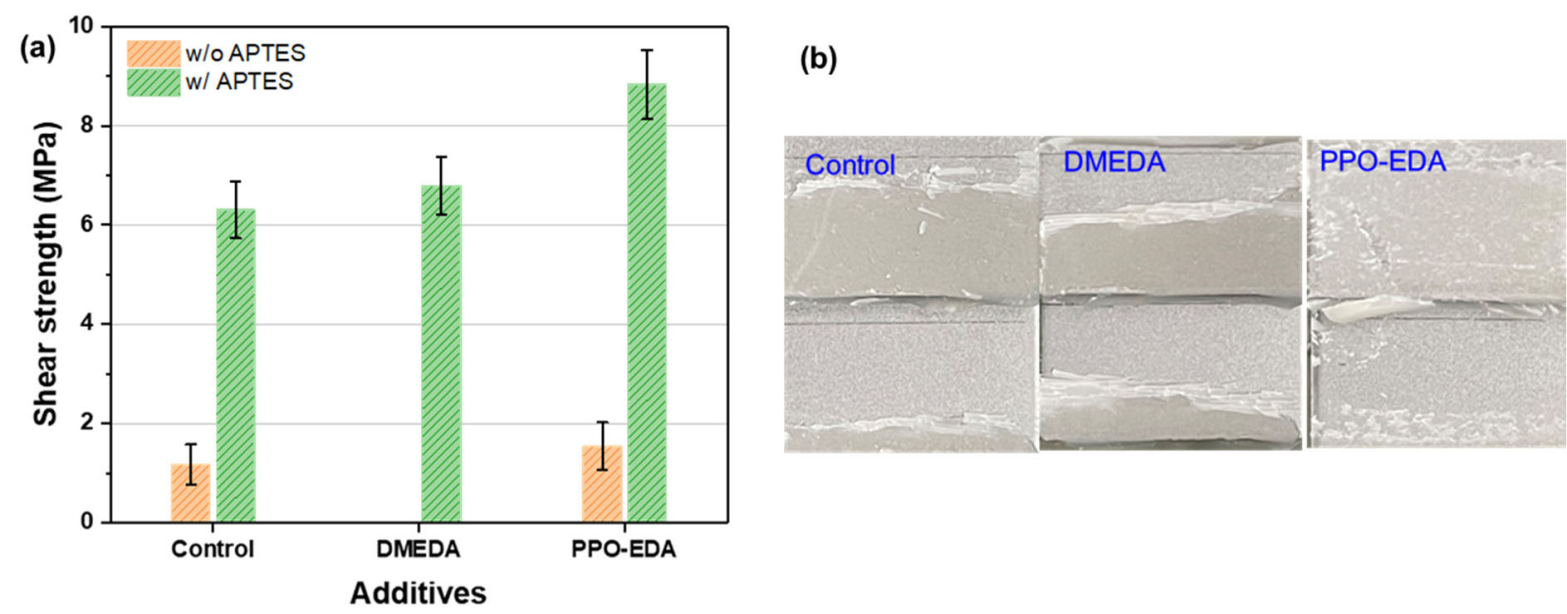
(**a**) Lap shear strength of PU adhesives on APS-treated and untreated steel substrates after 24 h of curing, and (**b**) images of the fractured surface of APS-treated specimens after lap shear testing.

To address this limitation, APS-treated steel substrates were used to reinforce interfacial adhesion. Figure 10 shows the lap shear strength of PU adhesives on APS-treated steel, highlighting the enhanced performance of the PPO-EDA-modified adhesive. On these treated substrates, the PPO-EDA-modified adhesives exhibit significantly higher lap shear strength compared to the control and DMEDA-modified adhesives. The failure mode shifts predominantly to cohesive failure, underscoring the improved cohesive strength contributed by PPO-EDA. This suggests that with APS treatment, lap shear values can effectively reflect the increased cohesion of PU adhesives due to interfacial reinforcement.

The differing lap shear strengths and failure modes observed with APS-treated substrates highlight the distinct roles of DMEDA and PPO-EDA in the PU adhesive matrix. DMEDA acts primarily as a chain extender, forming linear urea linkages that increase molecular weight without substantially increasing crosslink density. While DMEDA provides initial tensile strength, it lacks the structural integrity for sustained lap shear performance on untreated steel, leading to adhesive failure as curing progresses.

PPO-EDA, in contrast, functions as both a chain extender and a crosslinker due to its reactive NH and OH groups, which form urea and urethane bonds with NCO groups. This dual role significantly increases crosslink density, enhancing cohesion and stiffness within the adhesive matrix. On APS-treated substrates, the added polarity and cohesive energy from PPO-EDA promote stronger interfacial bonding through hydrogen bonding and van der Waals interactions, resulting in higher lap shear strength and dominant cohesive failure. Therefore, PU adhesives with PPO-EDA are expected to exhibit the highest cohesion, and the lap shear results align with this expectation.

However, on pristine steel substrates, the PPO-EDA modified adhesive does not achieve similarly high lap shear strength due to insufficient interfacial adhesion. Despite the increased cohesion within the adhesive layer, the lack of interfacial reinforcement limits performance improvements, leading to adhesive failure at maximum cure times. This underscores that when using pristine steel substrates, lap shear results do not capture the enhancements in bulk properties from additives like DMEDA and PPO-EDA due to interfacial failure.

## 4. Conclusions

This study demonstrated that PPO-EDA is an effective modifier for enhancing the mechanical and adhesive properties of PU adhesives. As an epoxide-modified diamine, PPO-EDA significantly increased NCO conversion, tensile strength, and crosslink density, while maintaining a balance between stiffness and flexibility. Compared to DMEDA, PPO-EDA acted as both a crosslinker and chain extender, resulting in a higher crosslinking density, which improved the elastic modulus and tensile strength of the adhesive. When amine silane-treated steel was used as the substrate, the lap shear strength followed the order of PPO-EDA > DMEDA > control, thus aligning with the tensile strength results. Even with small amounts of PPO-EDA, the PU adhesive exhibited high shear strength due to the increased crosslinking density, although tensile strain decreased from 33% to within 5%. While PPO-EDA improved lap shear strength, interfacial adhesion remained largely unchanged. In addition, our findings underscore the intricate balance between crosslinking and hydrogen bonding, which enhance rigidity, and the flexibility provided by aliphatic chains. This dynamic interplay plays a critical role in influencing glass transition temperature and mechanical performance, highlighting the importance of carefully balancing these opposing factors to fine-tune thermal transitions. Optimizing PPO-EDA content is essential to achieving maximum mechanical performance and adhesive properties in PU adhesives across diverse applications.

## Figures and Tables

**Table 1:** Detailed composition of two-component PU adhesives.

Composition		Control	DMEDA	PPO-EDA
Resin	Resin A	40.6	40.6	40.6
	DMEDA	-	0.2 (2.3 mmol)	-
	PPO-EDA	-	-	0.4 (2.3 mmol)
	Desiccant	5	5	5
	Filler (CaCO_3_)	54.4	54.4	54.4
Hardener	Resin B	100	100	100
	Filler (CaCO_3_)	24.0	24.0	24.0
NCO/OH		1.084		

**Table 2:** Yields of product and by-products with different conditions.

Yield * (Optimal)	Adding	Product (2 Adduct)	By-Product 1 (3 Adduct)	By-Product 2 (4 Adduct)
Bulk-25 °C	PPO	24.4	4.4	-
EtOH-25 °C	PPO	41.0	5.64	-
EtOH-25 °C	EDA	37.9	41.7	8.7

* Yields are calculated based on GC area percentage.

**Table 3:** Effect of additives on the glass transtion temperature, storage modulus, and crosslink density of PU adhesives.

Additive	*T_g_* (°C)	G′ at *T_g_* + 30 (MPa)	ν_e_ (×10^3^ mol/m^3^)
Control	37.7 ± 1.0	49.5 ± 3.9	17.5 ± 1.4
DMEDA	35.1 ± 1.1	54.8 ± 2.6	19.5 ± 0.9
PPO-EDA	35.8 ± 0.5	84.3 ± 7.7	29.9 ± 2.7

## Data Availability

The data are available from the authors on reasonable request.
